# Cross-cultural adaptation, reliability, and validity of a Chinese version of the pelvic girdle questionnaire

**DOI:** 10.1186/s12884-021-03962-8

**Published:** 2021-06-30

**Authors:** Hui Cong, Heng Liu, Yin Sun, Jinsong Gao, Juntao Liu, Liangkun Ma, Britt Stuge, Lixia Chen

**Affiliations:** 1 Department of Healthcare, Peking Union Medical College Hospital, Peking Union Medical College, Chinese Academy of Medical Sciences, Beijing, 100730 China; 2 Department of Orthopedics, Peking University First Hospital, Beijing, 100034 China; 3 Department of Obstetrics & Gynecology, Peking Union Medical College Hospital, Peking Union Medical College, Chinese Academy of Medical Sciences, Beijing, 100730 China; 4 Department of Orthopaedics, Oslo University Hospital, Kirkeveien 166, NO-0407 Oslo, Norway; 5 Department of Rehabilitation, Peking Union Medical College Hospital, Peking Union Medical College, Chinese Academy of Medical Sciences, No.1 Shuaifuyuan, Dongcheng District, Beijing, 100730 China

**Keywords:** Pelvic girdle pain, Pregnancy, Pelvic girdle questionnaire, Pain

## Abstract

**Background:**

The Pelvic Girdle Questionnaire (PGQ) is the only specific instrument designed to evaluate pain and activity limitations in pregnant or postpartum women with pelvic girdle pain (PGP). This study aimed to translate and culturally adapt the PGQ for Chinese patients and to verify the validation of the psychometric items of the PGQ in the Chinese population.

**Methods:**

First, the translation and cultural adaptation process of the PGQ was conducted on the basis of international guidelines. Eighteen women suffering from PGP (11 pregnant women and 7 postpartum women) were enrolled in the pilot tests. Second, a total of 130 pregnant and postpartum women with PGP were enrolled to evaluate the validation of the psychometric items of the Chinese version.

**Results:**

The calculated Cronbach’s alphas demonstrated a high level of internal consistency for the Chinese version of the PGQ, ranging from 0.77 to 0.93. The convergent validity showed a high positive correlation between the PGQ total score and the Oswestry Disability Index (0.84) and Numeric Rating Scale (0.73) for pain intensity. Furthermore, a good discriminatory ability was found for the Chinese version of the PGQ for distinguishing women who needed treatment from those not (area under the curve [AUC] = 0.843, *p* < 0.001), but not for discriminating the pregnant and postpartum states (AUC = 0.488, *p* = 0.824). The results of test–retest showed good reproducibility for the total PGQ (ICC = 0.93), the PGQ activity subscale (ICC = 0.92), and the PGQ symptom subscale (ICC = 0.77).

**Conclusion:**

Our study presents the translation, validation and psychometric features of the Chinese version of the PGQ, showing good construct validity and discriminative power for assessing the consequences of PGP among pregnant or postpartum Chinese women.

**Supplementary Information:**

The online version contains supplementary material available at 10.1186/s12884-021-03962-8.

## Background

Pelvic girdle pain (PGP) is a common pain during or after pregnancy, characterized by the presence of pain between the posterior iliac crest and the gluteal fold, particularly around the sacroiliac joints [1–3]. It is reported that approximately 45% of pregnant women and 25% of postpartum women suffer from PGP worldwide [4]. PGP severely interferes with the activities of daily living for pregnant and postpartum women [5]. The average sick leave for women with PGP during pregnancy or after pregnancy ranges from 7–12 weeks [6], resulting in huge economic costs. Thus, PGP should be given close attention in clinical practice.

The Oswestry disability index (ODI) and the Numeric Rating Scale (NRS) for pain intensity are the most common disability tools applied to evaluate PGP [7–11]. However, these questionnaires were originally developed for people with low back pain (LBP) and are not condition-specific measures for PGP [12, 13]. The Pelvic Girdle Questionnaire (PGQ) is the only instrument developed specifically for assessing PGP in pregnant or postpartum women [14–16]. Due to differences in language and cultural environment, patients from different countries will have different understandings of the PGQ items, leading to deviations in evaluation effectiveness [16, 17]. Therefore, it is best to translate the original PGQ into the corresponding language version as required. Currently, English, Norwegian, French, Iranian, Swedish, and Spanish versions of the PGQ are available [16–22].

As China is the most populous country in the world, a considerable number of pregnant women suffer from PGP every year in China. However, there is currently no standard treatment for these women in China. Thus, in many cases, timely and effective treatment cannot be given to women with PGP who really need treatment [23]. Previous studies have found that the original PGQ and the translated PGQ versions can effectively distinguish between PGP patients who need treatment and those who do not [17, 19]. However, to the best of our knowledge, a Chinese version of the PGQ that can be used in clinical practice has not been developed yet. Therefore, we conducted the present study to translate and culturally adapt the PGQ for Chinese patients and to assess its construct validity and discriminative ability in a Chinese population.

## Methods

**Fig. 1:**
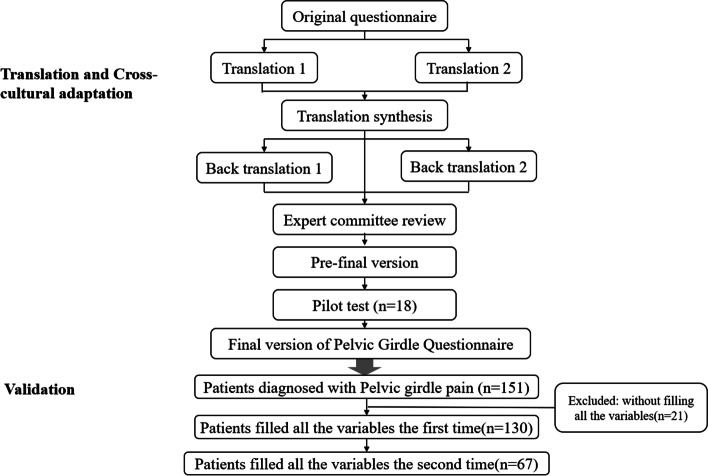
The flowchart of the study design

This research project was divided into two parts. In the initial part, the translation and cross-cultural adaptation procedures for the PGQ were conducted on the basis of international guidelines [24]. A total of 18 women with PGP were included in the pilot tests to assess the prefinal Chinese version of the PGQ in both pregnant women (*n* = 11) and postpartum women (*n* = 7). In the second part, a group of 130 pregnant or postpartum women suffering from PGP were used to validate the psychometric features of the Chinese version of the PGQ (Fig. 1). The Questionnaires were self-answered by all participants while in the hospital.

### Instruments

The PGQ is a simple instrument consisting of 25 items, including 20 items that assess activity limitations (activity subscale) and 5 items that evaluate symptoms (symptom subscale) [14]. Each item has 4 points and is scored using a Likert-type scale ranging from 0 to 3. The maximum possible score for the PGQ is 75, including 60 for the activity subscale and 15 for the symptom subscale. For an item without an answer or with a response of “not applicable”, three points are subtracted from the total possible score. The final data are presented as a percentage ranging from 0 (without disability) to 100 (severe disability). A higher score indicates a worse outcome.

The ODI (2.0 version) is one of the most commonly used tools in clinical practice for assessment of low back pain [11, 25]. Thus, we applied this instrument in our study to serve as a possible control in analyzing the relevance of the PGQ and ODI. This scale has 10 items, including pain intensity, personal care, lifting, walking, sitting, standing, sleeping, sexual life, social life and traveling. Each item has six response categories, ranging from 0 to 5 points [25]. The final data are divided into five categories (without disability, moderate disability, severe disability, crippled, and bedbound or exaggerating their symptoms) presented as a percentage score ranging from 0–100% with a 20% interval.

The 11-point NRS (0–10) is a tool commonly used to measure pain intensity [19]. It is labeled from 0 to 10, with 0 representing no pain and 10 reflecting the worst pain possible.

### Translation and cross-cultural adaptation

Approval was obtained from one of the authors of the original questionnaire before the translation process was started. The translation of the English version of the PGQ into Chinese was completed according to a 5-stage approach based on the guidelines for translation and cultural adaptation of questionnaires [26]. The detailed steps are as follows. The English version of the PGQ was independently translated into Chinese by two native Chinese speakers with a good command of English. Neither of the translators were previously familiar with the PGQ or PGP. One of the two translators was an orthopedic surgeon, and the other was a physiatrist. After translation, the two translated PGQs (Chinese versions) were compared with each other and synthesized, and then compared with the English version. Meanwhile, the synthesized Chinese version was conversely translated into English by two native English speakers independently. Both English translators, a professional translator and a physiotherapist, were not familiar with the original instrument before translation. After that, the two English translators were then introduced to the PGQ. A back-translation version (English) was obtained after an expert committee discussed the translations and resolved discrepancies. Experts proficient in psychometry, linguistics and PGP were included in this expert committee. For the purpose of capturing the accurate meaning of each item, the committee could refer back to the original Chinese version when difficulties were encountered in translations. A total of 18 women with PGP (11 pregnant women and 7 postpartum women) were enrolled to evaluate the prefinal version of the Chinese version of the PGQ. All the participants were required to explain the meaning of each point in the questionnaire and to describe any difficulty they experienced in understanding the question or statement. In addition, the participants were asked to reflect on the relevance of each item in the PGQ to their PGP situation. The women were asked to re-state the item if they encountered difficulties. Finally, all inconsistencies and difficulties were discussed until a consensus was reached by the expert committee.

### Validation

A total of 151 women suffering from PGP and attending our hospital were recruited for the second part of the study. All of the participants were clinically examined and confirmed to have PGP based on the recommendations from the European guidelines [24]. All the participants were enrolled from May 2019 to January 2020. Twenty-one participants were removed from the study because they did not complete the PGQ (missing items were found after the PGQ was collected). Finally, 130 participants were included in this study, and 67 of the included participants completed the PGQ again within 7 days. These data were used to evaluate the test–retest reliability. None of the patients enrolled in this study received any treatment for PGP.

The inclusion and exclusion criteria for the study were: PGP presenting in pregnancy or within 3 weeks after delivery; pain between the posterior iliac crest and the gluteal fold, particularly in the vicinity of the sacroiliac joint; pain radiating in the posterior thigh and also occurring in conjunction with/or separately from the symphysis; positive result on posterior pelvic pain provocation test and active straight leg raise test; positive result on the long dorsal sacroiliac ligament (LDL) test or symphysis pain palpation test, or positive for pain provocation of the symphysis by Modified Trendelenburg’s test; absence of hip disease, spondylolisthesis, lumbar disease or any autoimmune disease; no prior surgery related to the spine, pelvis or lower limbs; absence of radicular pain below the knee; absence of urinary disease; and absence of neurosensory disorders. Patients who met all the above criteria were diagnosed with PGP.

### Construct validity

The construct validity was calculated to test the extent to which scores on the Chinese version of the PGQ related to the theoretical concept of disability in women with PGP. The method reported by Annelie et al. was used to assess the divergent and convergent evidence of the construct validity [19], including five hypotheses: i) high correlation between the scores on the ODI and the activity subscale of the Chinese version of the PGQ; ii) moderate correlation between the scores on the ODI and the symptom subscale of the Chinese version of the PGQ; iii) high correlation between the scores for pain intensity and the total score on the Chinese version of the PGQ; iv) high correlation between the scores for pain intensity and the symptom subscale of the Chinese version of the PGQ; and v) moderate correlation between the scores on the NRS and the total score on the Chinese version of the PGQ.

### Discriminative validity

The area under the receiver operating characteristic (ROC) curve (AUC) was computed to evaluate the discriminative validity of the instrument for the following two aspects. First, the ROC curve was used to discriminate women with treatment or treatment needs reported in the instrument from women without treatment needs. Second, the ROC curve was applied to distinguish women during pregnancy from women in the postpartum period.

### Test–retest reliability

To further confirm the reliability of the questionnaire, 67 patients were asked to complete the questionnaire twice within 7 days. The intraclass correlation coefficient (ICC) was calculated to assess the sensitivity and reproducibility of the test. An ICC value greater than 0.80 indicated satisfactory reproducibility [27, 28]. The standard error of measurement (SEM) was also calculated based on the ICC value and its standard deviation using a previously reported formula [29, 30].

### Statistical analyses

Spearman’s correlation coefficients were computed to assess the association between the PGQ and ODI or NRS. Values less than 0.3 indicate low correlation; values from 0.3 to 0.6 indicate moderate correlation; and values greater than 0.6 indicate high correlation [19]. The internal consistency was computed using Cronbach’s alphas, and values ranging from 0.7 to 0.95 implied a good internal consistency. ROC curve analysis was performed to evaluate the discriminative ability of the Chinese PGQ. All statistical analyses were conducted using SPSS software (version 22.0, SPSS, Inc., Chicago, IL, USA). All results are presented as mean values with standard deviations.

### Ethical approval

All procedures conducted in this study involving human participants complied with the ethical standards of the Handbook for Good Clinical Research Practice of the World Health Organization and the Declaration of Helsinki principles (https://www.wma.net/policies-post/wma-declaration-of-helsinki-ethical-principles-for-medical-research-involving-human-subjects/).

## Results

### Translation and cross-cultural adaptation

The final analysis included 80 pregnant women (61.5%) and 50 postpartum women (38.5%). The mean age of these women was 32.2 years (SD 3.7 years), and the average gestational age in weeks was 29.7 (SD 8.1). One hundred twenty-five (96.2%) patients had a bachelor’s degree or higher. The detailed characteristics of the participants enrolled in the second part of this study are presented in Table 1. In general, most participants considered the items of the Chinese version of the PGQ to be concise, easy to understand, and strongly relevant to the symptoms of PGP. Two participants considered it hard to correctly understand the introductory text, the 16th item “Carry out sporting activities”, and the 19th item “Have a normal sex life”. Four participants had difficulties in correctly interpreting the meaning of the 23th item “Has your leg/have your legs given way”.

**Table 1:** Characteristics of participants enrolled in the validation portion (second part) of the study (*n* = 130)

Characteristic	
Pregnant	80(61.5%)
Gestational weeks	29.7(SD 8.1) (6–39)
Postpartum	50(38.5%)
Postpartum weeks	7.7(SD 5.4) (1–21)
Parity	
0	53(40.8%)
1	64(49.2%)
2	13(8.4%)
Age, years	32.2(SD 3.7) (23–40)
Body mass index, kg/m^2^	24.2(SD 2.5) (17.6–31.1)
Education	
High school diploma	5 (3.8%)
Bachelor’s degree	72 (55.4%)
Postgraduate degree	53 (40.8%)
PGQ total score	39.8 (SD 18.0) (5.3–81.9)
PGQ activity score	39.9 (SD 18.7) (5.0–84.2)
PGQ symptom score	39.8 (SD 17.4) (6.7–86.7)
ODI score	29.8 (SD 11.4) (8–56)
NRS score	3.9 (SD 1.8) (1–8)

*PGQ* Pelvic Girdle Questionnaire, *ODI* Oswestry Disability Index, *NRS* Numeric Rating Scale, *SD* standard deviation

### Validation

None of the tools included in this study showed ceiling or floor effects for either the total score or subscales scores of the PGQ. Eighteen women chose not applicable for the 16th item “Carry out sporting activities”, and 18 women chose not applicable for the 19th item “Have a normal sex life”. Thirty-four women did not provide an answer for the item about sex in the ODI.

**Fig. 2:**
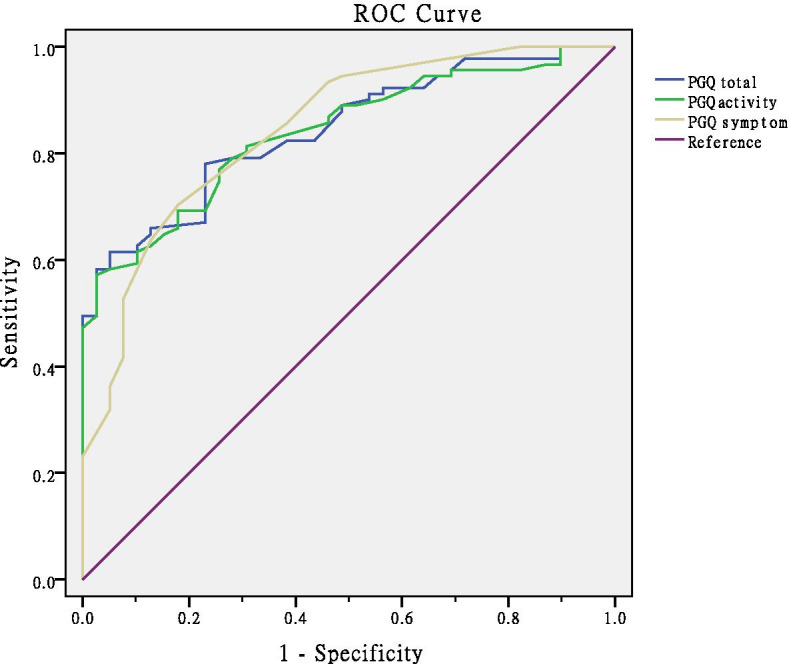
ROC curve analysis was used to evaluate the ability of the Chinese version of the PGQ to discriminate between treated women and non-treated women

The correlations among the Chinese version of the PGQ, ODI, and NRS for pain intensity are presented in detail in Table 2. The correlation coefficients for the relationships between the PGQ total score and ODI and NRS scores were 0.84 and 0.73, respectively. In addition, high correlations were found between the PGQ activity and symptom scores and the ODI score (0.82 and 0.71, respectively) and NRS score (0.80 and 0.72, respectively; Table 2). For the construct validity of the translated PGQ, five formulated hypotheses were evaluated, and four of them were confirmed: PGQ total to ODI, PGQ activity subscale to ODI, PGQ total to NRS, and PGQ symptom subscale to NRS (Table 3). For the discriminative validity of the translated PGQ, the results from ROC curve analysis showed that the Chinese version of the PGQ could significantly distinguish pregnant/postpartum women with PGP who received or requested treatment from those who do not need treatment (Fig. 2, Table 4). The AUC values for the PGQ total score, activity subscale score and symptom subscale score ranged from 0.772–0.918 (*p* < 0.001). The sensitivity and specificity of the translated PGQ for correctly predicting treatment and non-treatment were 0.57–0.70 and 0.82–0.97, respectively. However, the ROC curve analysis showed no significant discriminative ability of the PDQ for distinguishing pregnant women and postpartum women with PGP (Supplementary Table 1).

**Table 2:** Spearman’s correlation analysis among the pelvic girdle questionnaire, oswestry disability index and numeric rating scale (*n* = 130)

	PGQ total	PGQ activity	PGQ symptom	ODI	NRS
PGQ total	1				
PGQ activity	0.99	1			
PGQ symptom	0.89	0.84	1		
ODI	0.84	0.82	0.80	1	
NRS	0.73	0.71	0.72	0.67	1

*Note*: *PGQ* Pelvic Girdle Questionnaire, *ODI* Oswestry Disability Index, *NRS* Numeric Rating Scale

**Table 3:** Five a priori formulated hypotheses and correlation coefficients values for construct validity (*n* = 130)

Hypothesis	Instruments compared	Spearman’s correlation coefficient	Hypothesis confirmed?
High correlation between PGQ total score and ODI	PGQ total-ODI	0.84	Yes
High correlation between PGQ activity subscale and ODI	PGQ activity subscale-ODI	0.82	Yes
Moderate correlation between PGQ symptom subscale and ODI	PGQ symptom subscale-ODI	0.80	NO
High correlation between PGQ total score and NRS	PGQ total-NRS	0.73	Yes
High correlation between PGQ symptom subscale and NRS	PGQ symptom subscale-NRS	0.72	Yes

*Note*: *PGQ* Pelvic Girdle Questionnaire, *ODI* Oswestry Disability Index, *NRS* Numeric Rating Scale

**Table 4:** Comparison of instrument scores according to received or requested treatment by ROC curve analysis

	Value	n(%) of events	OR (95% CI)	Univariate logistic regression analysis *p*-value	AUC (95%CI)	*p* value
PGQ total score (OR per 5 units)	5- < 29 29–43 43 < 82	16 (40.0) 30 (68.2) 45 (97.8)	1.75 (1.40–2.20)	< 0.001	0.843(0.778–0.909)	< 0.001
PGQ activity score (OR per 5 units)	5- < 29 29–45 45- < 84	18 (40.0) 28 (71.8) 45 (97.8)	1.65 (1.35–2.02)	< 0.001	0.839(0.772–0.906)	< 0.001
PGQ symptom score (OR per 5 units)	6–30 30- < 40 40–86	13 (35.1) 30 (71.4) 48 (94.1)	1.79 (1.43–2.24)	< 0.001	0.845(0.772–0.918)	< 0.001

For the factor analysis, the Kaiser–Meyer–Olkin value was 0.824 and the p value of the Bartlett sphericity test was less than 0.001. A forced solution with three components was rotated using the Varimax method (Supplementary Table 2, Supplementary Figure 1).

### Test–retest reliability

Test–retest reliability was calculated with a random sub-sample of 67 patients who repeated the Chinese version of the PGQ after 1 week. The result of the internal consistency test showed satisfactory reproducibility (ICC > 0.77) for the total PGQ (ICC = 0.93), the PGQ activity subscale (ICC = 0.92), and the PGQ symptom subscale (ICC = 0.77) (Table 5). The SEM was 3.94%, indicating a low discrepancy between the results of a particular evaluation and the average of all the results hypothetically possible for a patient.

**Table 5:** Internal consistency for the instruments of the PGQ

	Number of women with missing data	All women, mean (SD)	Cronbach’s alpha
PGQ total			0.93
PGQ activity			0.92
1. Dress yourself	0	0.55(0.81)	
2. Stand for less than 10 min	0	0.43(0.70)	
3. Stand for more than 60 min	0	1.72(0.95)	
4. Bend down	0	1.60(0.89)	
5. Sit for less than 10 min	0	0.38(0.77)	
6. Sit for more than 60 min	0	1.50(0.97)	
7. Walk for less than 10 min	0	0.59(0.78)	
8. Walk for more than 60 min	0	1.79(0.91)	
9. Climb stairs	0	1.29(0.90)	
10. Do housework	0	1.22(0.84)	
11. Carry light objects	0	0.78(0.76)	
12. Carry heavy objects	0	2.10(0.85)	
13. Get up/sit down	0	1.10(0.96)	
14. Push a shopping cart	0	0.41(0.67)	
15. Run	0	1.72(1.01)	
16. Carry out sporting activities	18	1.84(.092)	
17. Lie down	0	0.98(0.98)	
18. Roll over in bed	0	1.72(1.06)	
19. Have a normal sex life	34	1.05(0.97)	
20. Push something with 1 foot	0	1.14(0.86)	
PGQ symptom			0.77
21. Pain in the morning	0	1.12(0.77)	
22. Pain in the evening	0	1.65(0.72)	
23. Has your leg/have your legs given way?	0	0.83(0.74)	
24. Do you do things more slowly?	0	1.52(0.75)	
25. Is your sleep interrupted?	0	0.85(0.81)	

*Note*: *PGQ* Pelvic Girdle Questionnaire

## Discussion

The aim of this study was to verify the transcultural adaptation and validation of the Chinese version of the PGQ. The results show that the Chinese version of the PGQ is easily accepted among women with PGP and presented good validation in terms of construction and discrimination of PGQ in China. Furthermore, good internal consistency and high reliability were verified for the Chinese version PGQ in the test–retest analysis.

The Chinese version of the PGQ shows similar construct validity to the original Norwegian version [22], as well as the translated Swedish version [19]. The present study shows lower internal consistency for the total scale compared with the Spanish translation (0.96) [17] and the Sweden translation (0.96) [19]. However, a consistency coefficient greater than 0.95 is considered indicative of redundancy among questionnaire items [27]. This demonstrates that all the items in the Chinese version of the PGQ are required as are those in the original Norwegian version.

In the study by Annelie et al., the Swedish version of the PGQ showed discriminatory ability between treatment and non-treatment pregnant/postpartum women with PGP [19]. Similarly, our results for discriminative validity indicate that the Chinese version of the PGQ could also effectively distinguish pregnant/postpartum women with PGP who need treatment from those who do not need treatment. This finding has important clinical implications for determining the need for intervention among Chinese women with PGP. This Chinese version of the PGQ can be used to effectively identify the PGP patients who need treatment in clinical practice. Thus, timely treatment can be given to such patients, avoiding continuous deterioration and improving their daily life [31, 32].

The original PGQ has been reported to have discriminatory power for separating pregnant women from postpartum women [22]. However, no significant difference was found in the AUC values for the translated PGQ for distinguishing between pregnant women and postpartum women with PGP, which is similar to the result for the Spanish version of the PGQ [17]. The possible reasons for this inconsistent result are the different educational backgrounds and parity of the patients [33]. The parity of most participants (91.6%) was 0 or 1 in the present study and 2 for only 13 women.

To confirm the reliability of the Chinese version of the PGQ, we performed a re-test of the PGQ on a random sub-sample of 67 patients who repeated the PGQ within 1 week. A high level of consistency was observed for the total score, the activity subscale score and the symptom subscale score, as indicated by the ICC values, representing the high reliability and stability of the Chinese version of the PGQ. Similar results were obtained for the Spanish version of the PGQ [17]. Further studies involved in translation of the PGQ are recommended to include the test–retest step in the validation to strengthen the evidence of the reliability of the translated PGQ.

Some limitations of this study should be considered. First, only 130 patients were included in this study to verify the cultural adaptation, reliability, and validity of the Chinese version of the PGQ, and most (96.2%) had a bachelor’s degree or a higher degree. Because most Chinese women only have a high school education [34], further research with a larger sample containing more women with only a high school education is required to confirm the reliability of the Chinese version of the PGQ for the general Chinese population. Second, the diagnosis of PGP in this study was mainly based on clinical tests. Because no statistical analysis was performed on the data from these clinical tests, we could not conduct a correlation analysis between clinical test results and scores on the Chinese version of PGQ. Finally, because only 151 women were included in this study, this study may have been underpowered from a statistical perspective.

## Conclusions

In summary, the Chinese version of the PGQ was found to be linguistically accurate, valid with high internal consistency, reliable and understandable for the evaluation of PGP disability among women both during and after pregnancy. The Chinese version of the PGQ showed a good ability to discriminate women who need treatment from women without a reported need for treatment. The use of this Chinese version of the PGQ will effectively improve the clinical care and research related to PGP in Chinese-speaking populations, promoting international research and decision-making based on common references.

## Supplementary Information

**Additional file 1: Supplementary Figure 1**. A scree plot showing distribution of factors by their eigenvalues.

**Additional file 2: Supplementary Table 1.** AUC value for the ability to discriminate between pregnancy and the post-partum state. **Supplementary Table 2.** Forced solution with three components and Rotated Total explained variance of Factor analysis.

## Data Availability

The datasets used and/or analysed during the current study are available from the corresponding author on reasonable request.

## Abbreviations

PGQ: Pelvic Girdle Questionnaire
PGP: Pelvic girdle pain
ICC: Intraclass correlation coefficient
SEM: Standard error of measurement
