# Supplementary figures and images for: Cross-cultural adaptation, reliability, and validity of a Chinese version of the pelvic girdle questionnaire
Source: BMC Pregnancy Childbirth. 2021 Jun 30;21:470. doi: 10.1186/s12884-021-03962-8 (PMC8247148; doi:10.1186/s12884-021-03962-8)

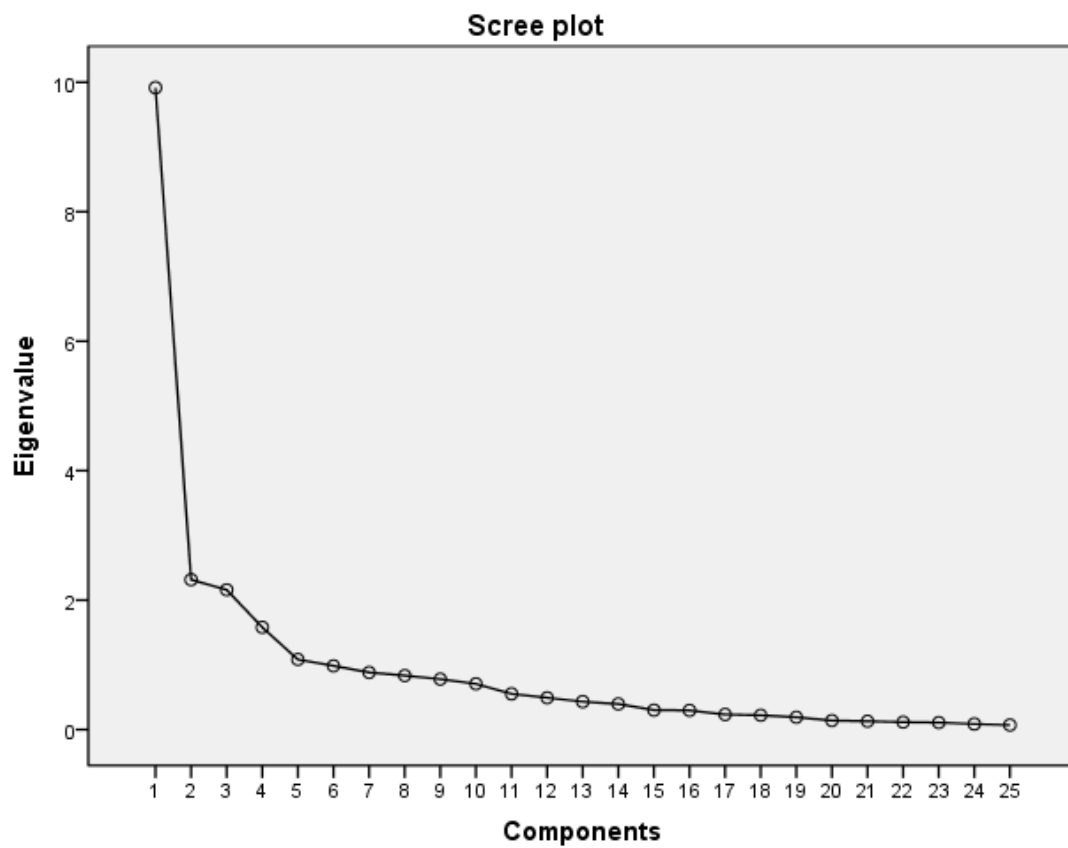

**Supplementary Figure 1:** A scree plot showing distribution of factors by their eigenvalues.

Supplement: Supplementary file 1 — Additional file 1: Supplementary Figure 1. A scree plot showing distribution of factors by their eigenvalues. [file 12884_2021_3962_MOESM1_ESM.pdf]
