# Supplementary material for: Cross-cultural adaptation, reliability, and validity of a Chinese version of the pelvic girdle questionnaire
Source: BMC Pregnancy Childbirth. 2021 Jun 30;21:470. doi: 10.1186/s12884-021-03962-8 (PMC8247148; doi:10.1186/s12884-021-03962-8)
Supplement: Supplementary file 2 — Additional file 2: Supplementary Table 1. AUC value for the ability to discriminate between pregnancy and the post-partum state. Supplementary Table 2. Forced solution with three components and Rotated Total explained variance of Factor analysis. [file 12884_2021_3962_MOESM2_ESM.pdf]

Supplementary Table 1. AUC value for the ability to discriminate between pregnancy and the post-partum state.

	p value	AUC (95% CI)
PGQ total score	0.824	0.488 (0.388-0.589)
PGQ activity score	0.765	0.484 (0.384-0.585)
PGQ symptom score	0.943	0.504 (0.403-0.605)

Supplementary Table 2. Forced solution with three components and Rotated Total explained variance of Factor analysis.

	Extractions sums of Squared loading			Rotation sums of Squared loading
Component	Total	Variance (%)	Accumulated (%)	Total
1	9.915	39.661	39.661	6.308
2	2.316	9.264	48.925	5.485
3	2.159	8.635	57.560	2.597
